# War injuries and civilian accidents in Afghan children

**DOI:** 10.1186/cc12216

**Published:** 2013-03-19

**Authors:** R Kedzierewicz, P Ramiara, M Puidupin, S Mérat

**Affiliations:** 1 CMA des Alpes, Barby, France; 2 HIA Sainte Anne, Toulon, France; 3 HIA Desgenettes, Lyon, France; 4 HIA Begin, Saint Mandé, France

## Introduction

We intended to compare paediatric traumatic injuries due to war and others related to civilian accidents (CA).

## Methods

We conducted an observational epidemiologic retrospective study on paediatric cases (<18 years old) seen at the emergency room of Kabul International Airport NATO role 3 medical treatment facility between 2009 and 2012.

## Results

During 3 years, 341 children were admitted to the emergency room (ER) with a mean age of 10 years (9.49 to 10.51). Eighty-eight per cent of children (301) were traumatized, 39% of them (118) due to war injuries (WI) and 61% (183) due to CA. Forty-three per cent of WI (gunshot wound 34%, explosion 66%) but only 19% of injuries due to CA (falls 24%, transport accidents 56%, burns 9%, penetrating injuries 9%, unknown cause 2%) involved polytraumas defined as a New Injury Severity Score (NISS) >15 (*P <*0.001). Patients experiencing a traumatic injury related to war had lower Paediatric Trauma Scores (PTS) than those injured in a CA (PTS = 6.4 (5.7 to 7.0) vs. PTS = 9.0 (8.7 to 9.4) respectively, *P <*0.00001), higher ISS (ISS = 16.7 (13.8 to 19.6) vs. ISS = 8.9 (7.5 to 10.2) respectively, *P <*0.00001), NISS (*P <*0.0001), mortality rate predicted by the Trauma Injury Severity Score (TRISS, *P <*0.02) or A Severity Characterization of Traumas (ASCOT, *P <*0.001). The result is longer overall hospitalization of patients having WI (*P <*0.001) and a higher number of surgeries (*P <*0.02). After the ER, 54% of patients with WI were hospitalized in the ICU (86% of them after surgery) but only 26% of patients involved in a CA (71% after surgery). As many patients with WI as involved in a CA (40%) were admitted to the ward (89% of patients with WI after surgery but only 63% of patients with injuries due to a CA). Thirty-three per cent of patients involved in a CA returned home and one was transferred, whereas only three patients with WI returned home after being in the ER, three patients were transferred and one died in the operating room. Observed paediatric mortality in our medical treatment facility was 2.9% (10 children out of 341): three children died of WI, three due to a CA and one of septic shock due to a medical cause.

## Conclusion

War injuries are more prone to cause polytrauma than CA. According to the PTS, ISS, NISS, TRISS and ASCOT, children experiencing WI have higher severity scores and predicted mortality rate than others, stay longer in the hospital and have more surgeries.

